# The Arc of Bühler: A Cadaveric Case Report of a Novel Variant

**DOI:** 10.7759/cureus.111826

**Published:** 2026-06-30

**Authors:** Ami Patel, Quan Dang, Cesar Ortiz-Alvarez, Michael Truong, Lily Liu, Jeremiah Scott

**Affiliations:** 1 Department of Medical Anatomical Sciences, Western University of Health Sciences, Pomona, USA

**Keywords:** arc of bühler, celiaco-mesenteric anastomosis, celiac trunk, inferior pancreaticoduodenal artery, superior mesenteric artery

## Abstract

The arc of Bühler (AOB) is a rare variant in the vasculature of the foregut and midgut. It forms as a direct anastomosis between the celiac trunk and the superior mesenteric artery or their branches. Here, we describe an unusually large AOB with a previously unreported pattern of anastomosis. The variant was discovered and documented during routine cadaveric dissection by first-year osteopathic medical students. The AOB arose from the celiac trunk and was similar in diameter to the splenic and common hepatic arteries. The AOB measured approximately 38 cm in total length along its pathway and terminated at the superior mesenteric artery. It varied in diameter from 4 mm to approximately 9-10 mm. Its connections to the surrounding viscera indicated that it supplied blood to the regions typically supplied by the right and middle colic arteries, which were not present as separate structures in the individual. A branch connected the AOB to the inferior pancreaticoduodenal artery at its origin from the posterior surface of the superior mesenteric artery. A review of the literature on AOB variants indicates that the pattern of branching reported here has not been described before. Patterns of anastomosis are clinically important because of their potential roles in pathophysiology (e.g., aneurysm, ischemia) and surgical procedures. Our findings highlight the importance of identifying this anatomical variant preoperatively and adjusting medical management accordingly, which can yield effective clinical results and potentially lower the risk of significant surgical complications in patients.

## Introduction

The vascular supply to the gastrointestinal tract and its accessory organs arises primarily from three major branches of the abdominal aorta [1]. The most superior branch, the celiac trunk, trifurcates to give off the left gastric artery, splenic artery, and common hepatic artery, which supply the foregut derivatives [2]. The second branch, the superior mesenteric artery (SMA), supplies the midgut derivatives via the jejunal, ileal, and colic arteries [1]. The third branch is the inferior mesenteric artery, which supplies the hindgut derivatives via the colic, sigmoid, and rectal arteries [1]. While this is the typical anatomy seen in the abdominopelvic viscera, anatomical variation in the vasculature is frequent and can include accessory or replaced arteries to solid organs, common stem abnormalities, and arterial shunts [3,4]. While vascular abnormalities are not rare, their undetected presence can lead to surgical complications, such as intraoperative bleeds, resulting in multiple attempts at hemostasis in regions where damage to more critical structures can occur [4].

Anastomoses between foregut and midgut vascular systems occur in three ways. The two main connections are the pancreaticoduodenal arcades linking the gastroduodenal artery and SMA, and the arcade of Kirk connecting the dorsal pancreatic artery to the SMA system via the middle colic artery or the pancreaticoduodenal arcades [2]. The third celiaco-mesenteric anastomosis is a rare variant known as the arc of Bühler (AOB), first described by Bühler and Tandler in 1904, with a pooled incidence rate of 1.71% (range 0.3-4.1%) from various studies [3]. When present, the AOB usually has a retro-pancreatic course, but its connections are variable [3]. The AOB can originate directly from the celiac trunk, making the trunk quadripartite, or indirectly from the splenic artery or its branches, common hepatic artery or its branches, or left gastric artery [3,5]. The anastomosis with the midgut vasculature is similarly variable, with the AOB connecting either directly to the SMA or indirectly via the middle colic artery, right colic artery, or inferior pancreaticoduodenal artery (IPDA), or their respective branches [3,5].

Here, we document an unusually large AOB with a previously unreported pattern of anastomosis, compare it to other variants previously reported in the literature, and discuss its potential clinical implications. Given its variable presentations, the AOB provides insight into the embryological processes that generate adult anatomy and presents an opportunity to improve interventional and surgical treatment methods, especially in cases of mesenteric ischemia, median arcuate ligament syndrome (MALS), and pancreaticoduodenectomies [6-9].

This article was previously presented as a poster at the Western Medical Research Conference, Carmel, California, on January 16, 2025 [10].

## Case presentation

**Figure 1:**
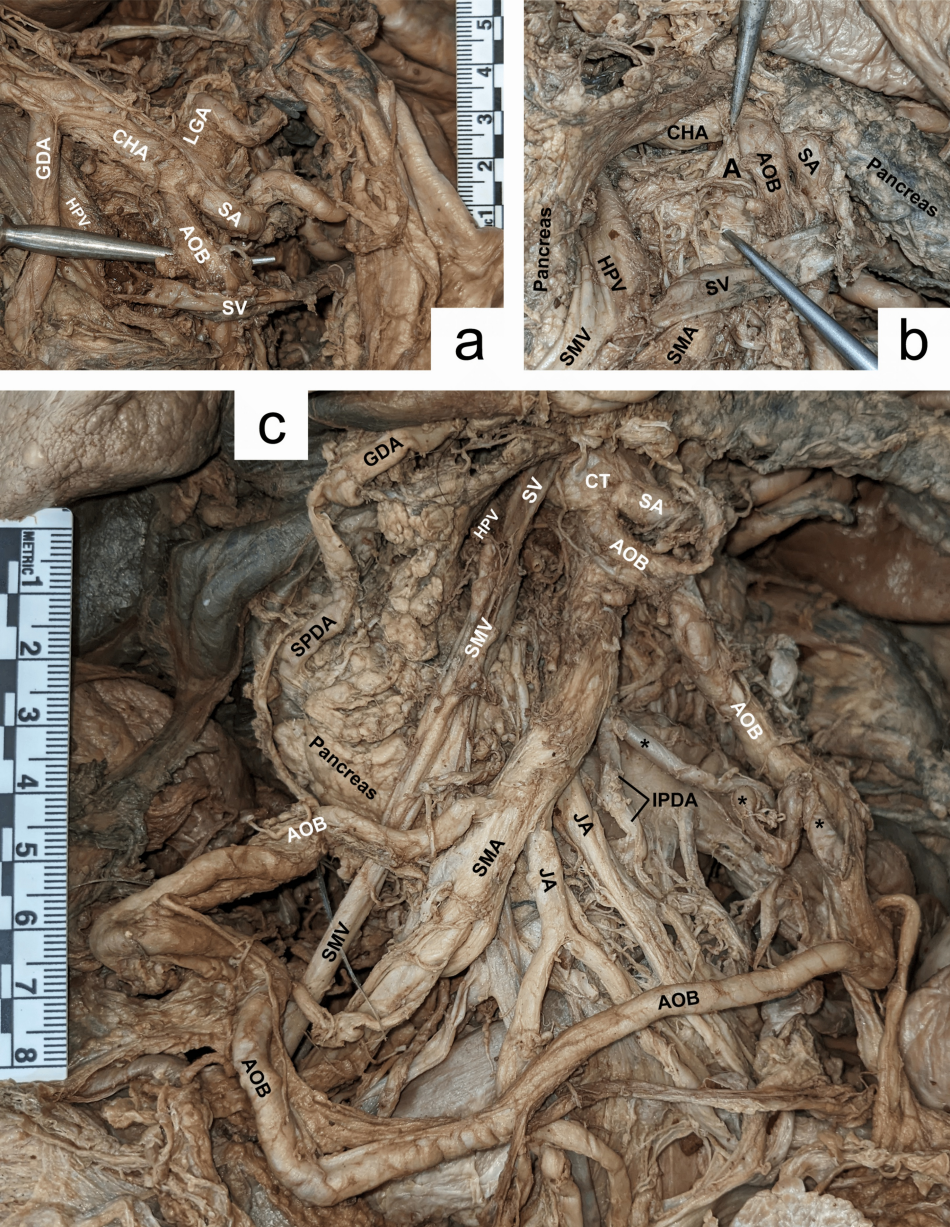
A novel variant of the AOB (a) The AOB, left gastric artery, splenic artery, and common hepatic artery emerging from the celiac trunk, which is located at the center of the four branches. (b) The celiac trunk has been reflected superiorly using a probe to reveal the abdominal aorta, partially obscured by the autonomic plexus; the lower probe marks the approximate origin of the SMA. The inferior vena cava is not visible; for reference, it is located posterior to the common hepatic artery and hepatic portal vein. (c) The full length of the AOB, from the celiac trunk to the SMA. The pancreas and splenic vein have been reflected superiorly; the transverse colon has been pulled inferiorly and is only visible at the lower right and left corners of the image. The branch of the AOB that connects with the IPDA is marked with asterisks. A, aorta; AOB, arc of Bühler; CHA, common hepatic artery; CT, celiac trunk; GDA, gastroduodenal artery; HPV, hepatic portal vein; IPDA, inferior pancreaticoduodenal artery; JA, jejunal arteries; LGA, left gastric artery; SA, splenic artery; SMA, superior mesenteric artery; SMV, superior mesenteric vein; SPDA, superior pancreaticoduodenal artery; SV, splenic vein.

A large vessel arising from the inferior part of the celiac trunk, along with the left gastric, splenic, and common hepatic arteries, was found during routine dissection by first-year osteopathic medical students at the Western University of Health Sciences, Pomona, CA (Figures 1a, 1b). The embalmed body donor was an 83-year-old male with a history of hypertensive heart disease, aortic atherosclerosis, interstitial lung disease, and sick sinus syndrome. The vessel was subsequently identified as an AOB based on its anastomoses. The AOB measured 7-8 mm in diameter at the celiac trunk (all diameters reported here were measured by positioning the scale bar underneath the artery; they should be considered approximate). The vessel followed a tortuous pathway from its origin at the celiac trunk (Figure 1c). It was traced distally to the left side of the transverse colon. It then ran alongside the transverse colon to the superior part of the ascending colon and right colic flexure, giving off short branches to supply regions normally perfused by the middle and right colic arteries. The latter two arteries were not present in their usual form. The AOB then terminated at the SMA. After dissection and mobilization, we measured the path length of the vessel using a string. The total traced length of the AOB was approximately 38 cm from the celiac trunk to the SMA. The maximum and minimum diameters were approximately 9-10 mm and 4 mm, respectively. The minimum diameter occurred at the junction with the SMA.

**Figure 2:**
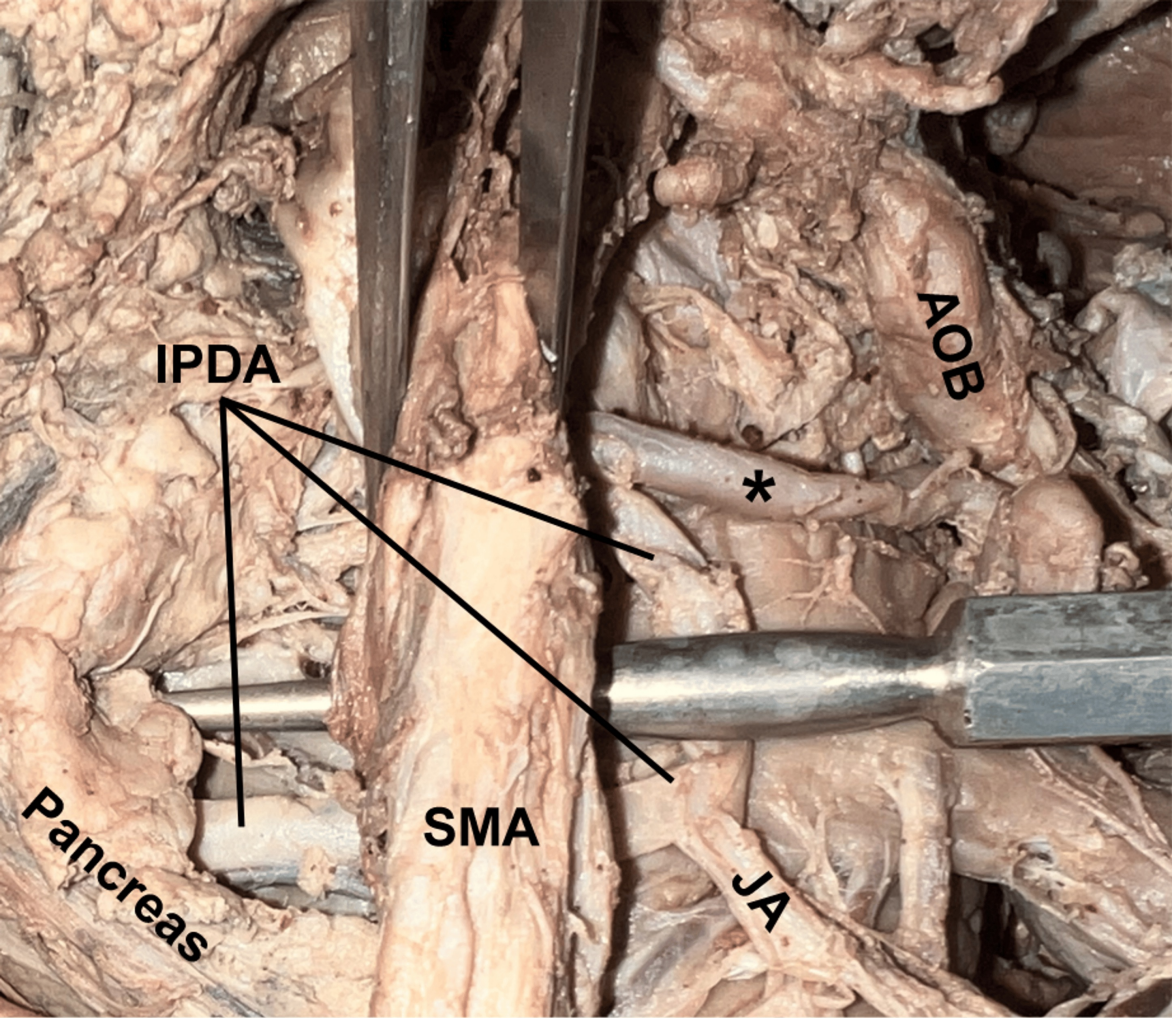
Close-up of the anastomosis with the IPDA The branch of the AOB that connects to the IPDA at the latter’s origin from the SMA is marked with an asterisk. The IPDA runs posterior to the SMA before entering the pancreas. AOB, arc of Bühler; IPDA, inferior pancreaticoduodenal artery; JA, jejunal artery; SMA, superior mesenteric artery

The AOB also gave rise to a branch that connected to the IPDA, which originated from the posterior surface of the SMA. The branch from the AOB connected to the IPDA at the IPDA’s origin from the SMA, forming a tripartite junction (Figure 2). After emerging from the SMA, the IPDA turned right and ran posterior to the SMA, entering the head of the pancreas on its posterior surface. There was no evidence that the IPDA bifurcated into anterior and posterior branches, which typically anastomose with their counterparts descending from the superior pancreaticoduodenal artery. The IPDA gave off a jejunal branch prior to turning right to cross behind the SMA. The origin of the IPDA is variable; the origin documented in this case has been reported previously [11].

## Discussion

**Figure 3:**
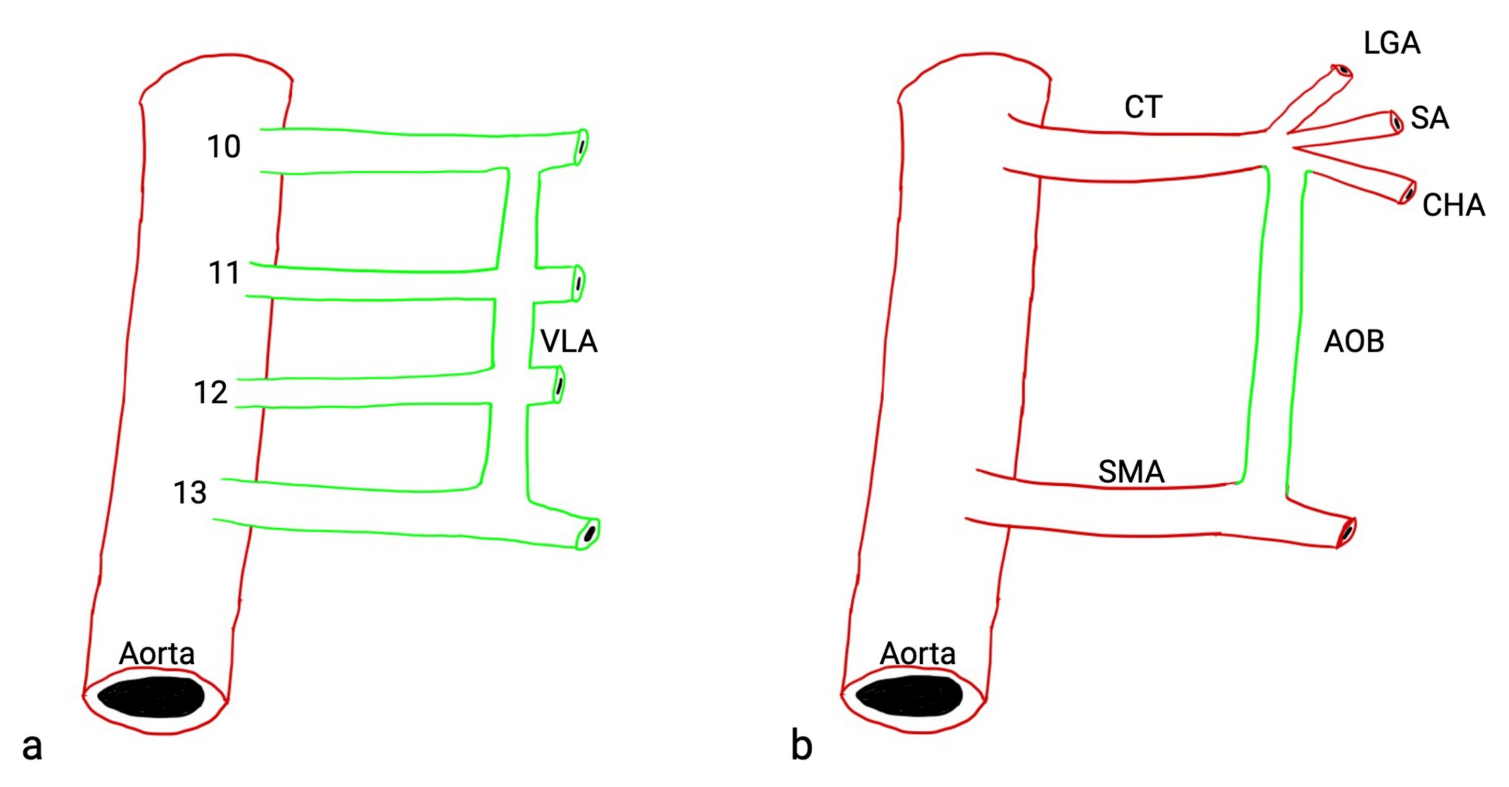
Embryological origin of the AOB A schematic of the embryological origin of the AOB, as explained by Tandler’s theory of VLA. (a) The 10th–13th segmental branches emerging from the aorta, connected by the VLA. Under normal embryological development, the 10th, 11th, and 12th segmental branches will fuse together to form the celiac trunk and its three branches: LGA, SA, and CHA, respectively. The 13th segmental branch will give rise to the SMA, and the VLA will regress. (b) Normal fusion pattern of the 10th, 11th, and 12th segmental branches but with the persistence of the VLA, which forms the AOB. AOB, arc of Bühler; CHA, common hepatic artery; LGA, left gastric artery; VLA, ventral longitudinal anastomosis; SA, splenic artery; SMA, superior mesenteric artery This image is based on illustrations presented in Douard et al. [13] and was created by Ami Patel using the Goodnotes app (Goodnotes Limited, London, UK).

Tandler’s theory of ventral longitudinal anastomosis provides a framework for understanding anatomical variation in the branches of the abdominal aorta [12]. Early in embryological development, the 10th through 13th ventral segmental arteries branch from the abdominal aorta and are united by a ventral longitudinal anastomosis (Figure 3). As the embryo develops, these ventral segmental arteries and the longitudinal anastomosis will migrate, fuse, or regress as their definitive organs reach their final anatomical placement [12,13]. The celiac trunk is formed by the fusion of the 10th through 12th ventral segmental arteries that develop into the left gastric artery, splenic artery, and common hepatic artery, respectively. The 13th ventral segmental artery persists as the SMA [12,13]. While most anatomical variations in mesenteric vasculature can be explained by varying degrees of development of these segmental arteries, it is the complete or partial failed regression of the longitudinal anastomosis that leads to a persistent direct anastomosis between the celiac trunk and the SMA, or their branches, known as the AOB.

**Figure 4:**
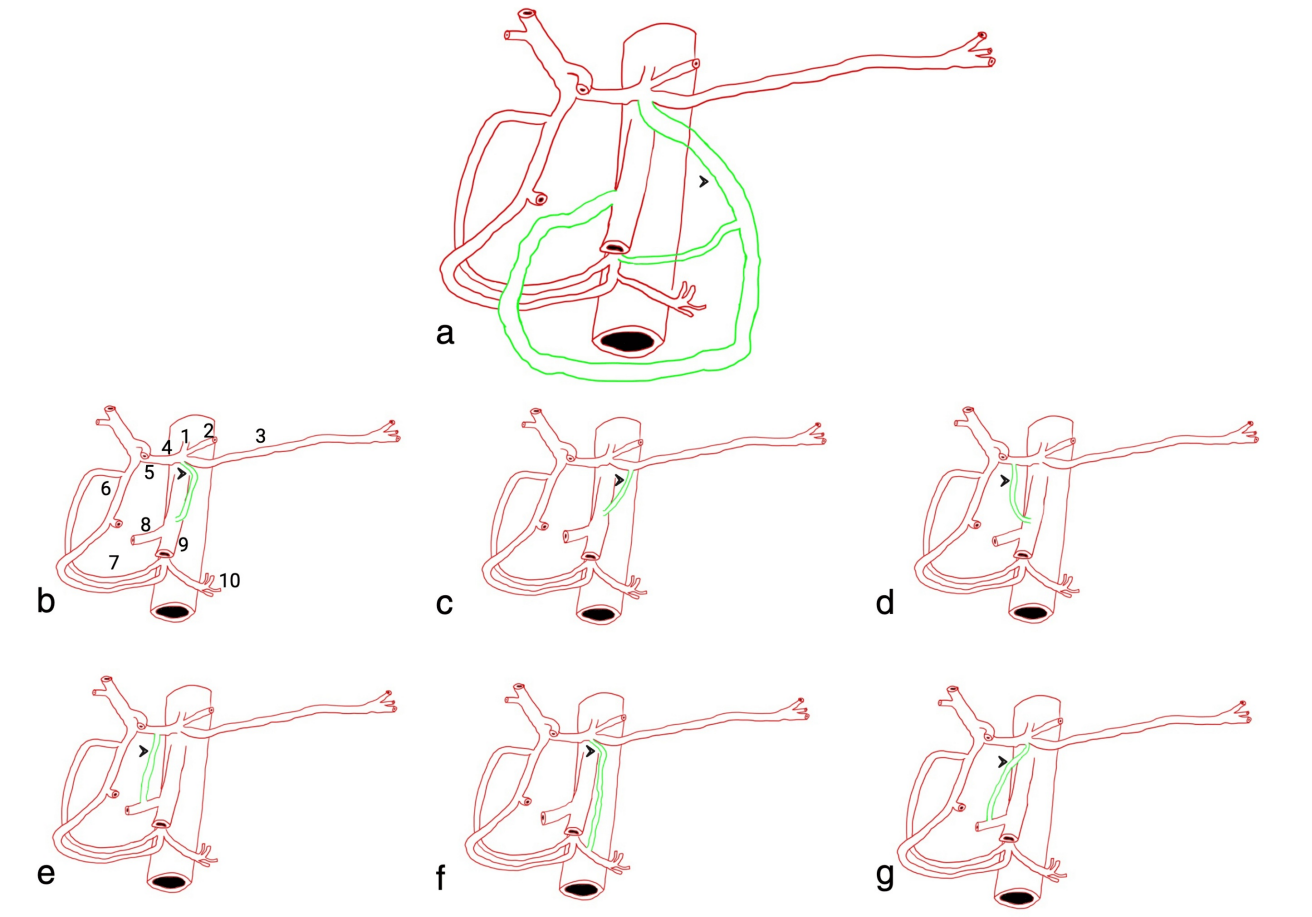
Previously described variants of the AOB compared to the new variant A schematic of the novel AOB variant (a) and previously reported variants (b–g). In each diagram, the AOB is the green vessel indicated by the arrowhead. Numbers indicate the following: (1) celiac trunk, (2) left gastric artery, (3) splenic artery, (4) common hepatic artery, (5) gastroduodenal artery, (6) superior pancreaticoduodenal arteries, (7) inferior pancreaticoduodenal arteries, (8) middle colic artery, (9) superior mesenteric artery, (10) first jejunal artery. AOB, arc of Bühler This image is based on an illustration presented in Michalinos et al. [3] and was created by Ami Patel using the Goodnotes app (Goodnotes Limited, London, UK).

Several AOB variants have been noted previously, but our review of the literature indicates that the pattern of anastomosis described here, with the AOB connecting the celiac trunk directly to both the SMA and IPDA (Figure 4a), has not been reported previously [2,3,5,14-17]. The literature review consisted of a thorough search of PubMed and Google Scholar using the following search terms: “Arc of Bühler”, “celiac trunk variations”, “celiac trunk anastomoses with SMA”, “Arc of Bühler aneurysms”, and “Arc of Bühler embryology”. The AOB is known to directly connect the celiac trunk with the SMA (Figure 4b), the splenic artery with the SMA (Figure 4c), the common hepatic artery with the SMA (Figure 4d), the common hepatic artery with the middle colic artery (Figure 4e), the celiac trunk with the first jejunal artery (Figure 4f), and the celiac trunk with the middle colic artery (Figure 4g) [5,14-16]. Additionally, the AOB has also been reported to give off branches, such as a mesenterico-jejunal branch, jejunal artery, or a variation of both [2,14,17]. The variant described here fully replaced the middle colic and right colic arteries, which were not present as separate structures in the individual. We did not find a variant of this nature in the literature [2,3].

The presence of an AOB is generally asymptomatic and detected largely incidentally on computed tomography angiography (CTA) when investigating other conditions. Whether or not the AOB plays a hemodynamically significant role in mesenteric blood flow is dependent on the diameter of the patent vessel, as quantified by Saad et al. using a 1.67-mm catheter [18]. Saad et al. used digital subtraction contrast angiography to measure the diameter and preferential filling of the celiac trunk via the AOB when power-injecting the SMA, and vice versa. They found that an AOB diameter of less than 1.5 mm was not sufficient to opacify the celiac trunk or its branches, whereas an AOB diameter between 1.5 and 2.5 mm allowed the anastomosis to act as a collateral vessel between the celiac trunk and SMA in cases of arterial occlusion [18]. With an external diameter ranging between 4 mm and 10 mm, the large AOB presented here suggests significant potential for influencing the continuity of blood flow and perfusion within the mesenteric vasculature, which may have contributed to the individual’s existing conditions in potentially protective or harmful aspects. We note, however, that we have only limited information about the individual’s medical history, precluding an informed discussion of the AOB variant’s possible effects in this particular case.

When hemodynamically significant, the AOB can play a protective role in individuals with mesenteric ischemia, providing an alternate route for blood flow in cases of occlusion or external compression of either the celiac axis or SMA [19]. This protective role has been documented in several cases. For example, O’Brien and Ferral presented a case of MALS where the presence of an AOB appeared to have an influence on the manifestation of symptoms in a 70-year-old patient. In MALS, the median arcuate ligament compresses the celiac axis, causing chronic mesenteric ischemia and severe upper abdominal pain, nausea, diarrhea, and sweats. While MALS typically requires surgical and/or endovascular intervention in younger adults, the 70-year-old patient had gradual, complete occlusion of his celiac artery and stenosis of the SMA yet had a late diagnosis and only presented with mild symptoms [9]. Furthermore, high-grade celiac stenosis due to MALS was found to be asymptomatic preoperatively due to the collateral flow from a persistent AOB in an individual undergoing a pancreaticoduodenectomy for a pancreatic mass [6]. In the case of pancreaticoduodenectomy, resection of the gastroduodenal artery eliminates an avenue of collateral blood flow between the celiac trunk and SMA (i.e., between the superior and inferior pancreaticoduodenal arteries). However, Schumacher et al. found that no stent or arterial bypass was required in a patient with a persisting AOB discovered preoperatively or intraoperatively [8]. In this case, the presence of the AOB provided collateral blood flow, preventing potential liver ischemia and complex surgical procedures or complications. When an AOB found intraoperatively was resected in a pancreaticogastrostomy case, abdominal pain, gastric ischemia, and necrosis of the remaining pancreas ensued, requiring additional surgical interventions [8]. Furthermore, a 54-year-old man with a type B aortic dissection and dissected celiac trunk that led to a suspected splenic infarct was spared a bypass surgery of the celiac trunk because blood flow to foregut viscera was maintained through the incidental discovery of a patent AOB during urgent CTA. The patient was spared the increased risks associated with a celiac artery bypass surgery, needing only a stent to address the type B aortic dissection [7]. These cases emphasize the significance of evaluating for collateral pathways in cases of mesenteric vascular ischemia, stenosis, occlusion, or surgery and exemplify the protective benefits a patent AOB can have in reducing the severity of symptoms and prolonging the asymptomatic period for such conditions.

While an AOB presents beneficial properties, it can also lead to aneurysm development. Aneurysms are the most significant pathology of an AOB, and most are found incidentally or unruptured and treated prophylactically with surgical resection or coil embolization, with no reported morbidity or mortality resulting from these procedures [3,20]. These visceral artery aneurysms occurred due to increased blood flow from a celiac trunk or SMA occlusion, causing the AOB to become dilated as a collateral vessel, inducing fibromuscular, atherosclerotic, and fibroblastic changes in arterial wall histology, as was the case of a 59-year-old Japanese woman, resulting in surgical removal of the AOB aneurysm after a failed transarterial embolization [16]. Such aneurysms can also increase the risk of clot formation, which potentially predisposes the patient to a higher risk of arterial occlusion and ischemia of abdominal visceral organs [3]. While these risks are extremely rare, clinicians and surgeons must be aware of this anatomical variation, further emphasizing the importance of accurate mapping of the abdominal vasculature to maximize preventative measures.

## Conclusions

The diversity and complexity of the celiaco-mesenteric anastomoses highlight the plasticity of embryonic ventral segmental and longitudinal vessels during development. The various manifestations of the AOB are significant for clinical diagnoses, their outcomes, and surgical procedures associated with abdominal viscera. The novel aspects of the AOB reported here, including its length, diameter, and branching pattern, enhance our knowledge of clinically important anatomical variants. The structure of the anastomosis also emphasizes the need to recognize vascular variants during preoperative planning to guide operative approaches, mitigate potential surgical complications, and improve patient outcomes.
